# Adult first-generation immigrants and cardiovascular risk factors in the Veneto Region, Northeast Italy

**DOI:** 10.3389/fpubh.2023.956146

**Published:** 2023-02-15

**Authors:** Teresa Dalla Zuanna, Erich Batzella, Gisella Pitter, Francesca Russo, Teresa Spadea, Cristina Canova

**Affiliations:** ^1^Unit of Biostatistics, Epidemiology and Public Health, Department of Cardio-Thoraco-Vascular Sciences and Public Health, University of Padua, Padova, Italy; ^2^Screening and Health Impact Assessment Unit, Azienda Zero, Padova, Italy; ^3^Directorate of Prevention, Food Safety, and Veterinary Public Health-Veneto Region, Venice, Italy; ^4^Epidemiology Unit ASL TO3, Turin, Italy

**Keywords:** immigrants, cardiovascular risk (CV risk), cholesterol, blood pressure, hypertension, acculturation

## Abstract

**Introduction:**

The health condition of immigrants traditionally follows a transition from a low disease occurrence to the epidemiological profile of the deprived groups in the host country. In the Europe, studies examining differences in biochemical and clinical outcomes among immigrants and natives are lacking. We examined differences in cardiovascular risk factors between first-generation immigrants and Italians, and how migration pattern variables could affect health outcomes.

**Material and methods:**

We included participants between 20 and 69 years recruited from a Health Surveillance Program of the Veneto Region. Blood pressure (BP), total cholesterol (TC) and LDL cholesterol levels were measured. Immigrant status was defined by being born in a high migratory pressure country (HMPC) and subdivided by geographical macro-areas. We used generalized linear regression models to investigate differences between these outcomes among immigrants compared to native-born, adjusting for age, sex, education, BMI, alcohol consumption, smoking status, food consumption, salt consumption in the BP analysis and the laboratory in charge for cholesterol analysis. Within immigrant subjects, the results were stratified by variables of the migration pattern: age at immigration and length of residence in Italy.

**Results:**

Thirty seven thousand three hundred and eighty subjects were included in the analysis, 8.6% were born in an HMPC. Heterogeneous results were seen by the macro-areas of origin and sex, with male immigrants from CE Europe (β = 8.77 mg/dl) and Asia (β = 6.56 mg/dl) showing higher levels of TC than native-born, while female immigrants from Northern Africa showed lower levels of TC (β = −8.64 mg/dl). BP levels were generally lower among immigrants. Immigrants residing in Italy for more than 20 years had lower levels of TC (β = −2.9 mg/dl) than native-born. In contrast, immigrants who arrived <20 years ago or arrived older than 18 years had higher levels of TC. This trend was confirmed for CE Europeans and was inverted for Northern Africans.

**Conclusions:**

The large heterogeneity in the results depending on sex and macro-area of origin indicates the need for targeted intervention in each specific immigrant group. The results confirm that acculturation leads to a convergence toward the epidemiological profile of the host population that depends on the starting condition of the immigrant group.

## 1. Introduction

The unprecedented flow of immigrants toward Europe in the last few decades has turned most European countries into multiethnic societies. Italy, like other South European countries, has experienced a relevant increase in this phenomenon in the last 20 years, with the percentage of the immigrant population reaching 8.5% in 2018 (1). The health condition of immigrants in comparison to natives is traditionally expected to follow a transition from a low disease occurrence in the first period after arrival (so-called “healthy migrant effect”) (2) to a progressive convergence toward the health behaviors and epidemiological profile of the lowest socioeconomic groups of the host population (3). This acculturation process, which entails an increase in risky behaviors (4) and the adoption of a Westernized diet and a more sedentary lifestyle (5), represents a threat to their physical and mental health. Furthermore, the accessibility of health services for immigrants is often undermined by cultural and language barriers, creating new challenges for health systems.

In Europe, limited information is available on ethnic differences concerning biochemical and clinical parameters known as CVD risk factors, such as elevated plasma lipid levels and elevated blood pressure levels (6, 7). Most of the data are derived from comparisons between studies conducted in the United States (8, 9). A study conducted in the Netherlands found large ethnic differences in lipid components, both in unadjusted models and in models adjusted for multiple covariates known to affect lipid metabolism. These results suggested that, next to lifestyle factors, intrinsic differences in lipid metabolism may contribute to the observed differences in plasma lipid levels (10). A review on ethnic differences in blood pressure levels in Europe found higher blood pressure levels in immigrants from Sub-Saharan Africa over decades and lower levels in the Muslim population, suggesting the limited efficacy of prevention in some groups and that untapped lifestyle and behavioral habits may reveal advantages toward the development of hypertension (11).

In Italy, studies based on health administrative data or nationwide surveys are improving the knowledge concerning the health status of immigrant populations compared to native-born Italians. The differential disease prevalence has been widely studied, it has been shown that diabetes mellitus prevalence is higher among immigrants than among native-born Italians (12). The prevalence of other cardiovascular diseases is comparable, showing heterogeneous patterns for immigrants from different countries of origin but with worse indicators of the clinical management of the disease (12). On the other hand, studies examining differences in biochemical and clinical outcomes, known as cardiovascular risk factors, among immigrants and natives are lacking. Increased levels of total and LDL cholesterol and high blood pressure are the most prevalent conditions increasing the risk of cardiovascular disease (CVD) (13). Since biochemical alterations are detectable before the onset of the disease itself, we focused the analysis on these risk factors that could help in detecting health differences earlier than the analyses on the confirmed diseases. Early detection of these differences, especially in young populations, could provide more chances for early treatments and reduce future health inequalities.

The objective of this study is to examine the differences in the lipid profiles and blood pressure levels between first-generation immigrants and native-born Italians in a large population of the Veneto Region, Northeast Italy. This piece of information could provide insight into understanding the mechanisms behind emerging differences in the occurrence of cardiovascular diseases. The immigrant population is very heterogeneous and should possibly be analyzed considering differences within this category. Therefore, we also analyzed these data by geographical macro-area of origin to investigate how variables of the migration pathway could affect health outcomes.

## 2. Material and methods

### 2.1. Participants and study design

We analyzed data from a publicly funded health surveillance program implemented by the Veneto Region in 30 municipalities in this Region, located in Northeast Italy (14). This program is a population-based screening program with the aim of the prevention, early diagnosis, and treatment of chronic disorders possibly associated with the high perfluoroalkyl substances exposure–PFAS, manufactured chemicals with grease-, stain-, and water-repelling properties–that was discovered in this area in 2013. The program started in 2017, and it is still ongoing, with no cost for participants. The target population included 105,000 residents of the contaminated area born between 1951 and 2014. Eligible subjects were identified through the regional health registry, which contains personal and residency data for the entire population of the Veneto Region. Residents who decided to participate in the program completed a structured interview administered by a trained public health nurse, followed by blood pressure measurement and blood and urine sampling. Program visits were performed at public health facilities located throughout the contaminated area to ensure easy accessibility. Data were collected using centralized web-based software connected with the regional health registry. The software allows the extraction of lists of eligible residents, online compiling of interview and blood pressure data, and retrieval of laboratory test results. To maximize data quality by minimizing errors and missing values, standard data checks and cleaning procedures (e.g., range and consistency checks) were performed.

All recruited subjects until May 2021, aged between 20 and 69 years old, were included in this analysis (*n* = 38,292, participation rate 61%). Pregnant women at the time of participation in the study and participants with missing data on relevant variables were excluded, leaving 37,710 subjects included in the analysis (Supplementary Figure S1). No missing data on exposure and outcome variables were present.

### 2.2. Outcome assessment

Nonfasting blood and urine samples collected from participants were sent to three local health unit laboratories within the study area (Arzignano, San Bonifacio, Legnago). Blood pressure was measured according to the European Society of Hypertension recommendations (15).

The outcome variables include the following:

- Blood lipids (total cholesterol = TC, and LDL cholesterol = LDL-C, calculated using Friedewald formula).
- Systolic and diastolic blood pressure (SBP, DBP).
- Hypertension, defined considering any self-reported diagnosis of hypertension, reported use of antihypertensive medications, or SBP≥140 mmHg or DBP≥90 mmHg.

### 2.3. Exposure: Country of birth and residential history

Immigrant status was defined as the country of birth reported by the participants. Immigrants born in high migratory pressure countries (HMPCs) were further grouped into 5 geographical macro-areas of origin: Central-Eastern (CE) Europe, Central and Southern (CS) America, North Africa, Sub-Saharan (SS) Africa and Asia (except for Israel and Japan). Immigrants born in highly developed countries (HDC) were a very small percentage (330, 0.87% of the study population) and were excluded from this study (Supplementary Figure S1).

Each participant of the surveillance program was asked about his or her residential history, including all episodes of transfer of residence to a different country/municipality. The year of immigration to Italy was calculated for immigrants from HMPC countries as the first date of residence in any Italian municipality. The age at immigration (categorized as ≥18 years old and <18 years old, as it is in Italy the age of majority) and length of residence were subsequently calculated. The latter was categorized as <10 years, between 10 and 19 years and ≥20 years, because of the distribution of our population and the need of a sufficient statistical power. Subjects with missing information on residential history, as well as subjects with nonlinear migration pathways—such as long returns to their country of origin—were excluded from these analyses (Supplementary Figure S1).

### 2.4. Covariates

The following range of potential confounders were considered based on prior literature: age (years), sex, education [primary/middle school, high school, university or higher), BMI (from self-reported height and weight and classified as normal weight (<25), overweight (25–29.9), or obese (≥30)], alcohol consumption (0, 1–2, or 3+ alcohol units per week), smoking status (current smokers, previous smokers, or nonsmokers), and food consumption. Data on food consumption (meat, fish/seafood, milk/yogurt, cheese, eggs, bread/pasta/cereals, sweets/snacks/sweet beverages, fruits/vegetables) were transformed from the number of servings per day/week/month to the number of servings per week and categorized into tertiles or quartiles to allow a harmonized diet pattern classification. Furthermore, salt consumption (categorized as low, medium, or high) was considered a possible covariate in the blood pressure analyses. Finally, information on the laboratory in charge of the analyses of serum lipids (Arzignano, Legnago, and San Bonifacio) was considered a possible confounder in the statistical analyses of cholesterol levels.

### 2.5. Statistical analysis

First, the demographic and lifestyle characteristics of HMPC-born residents and natives were compared.

Participants who had reported using cholesterol-lowering medications such as statins, fibrates and red rice were excluded for serum lipid outcomes (*n* = 1,676), and participants with a self-reported diagnosis of hypertension or under treatment with antihypertensive medications were excluded for continuous blood pressure outcomes (*n* = 4,859), leaving 35,704 and 32,521 subjects included for the lipid and blood pressure analyses, respectively (Supplementary Figure S1).

We used generalized linear regression models (LMs) to investigate the differences between cardiometabolic outcomes (SBP, DBP, TC and LDL-C) among HMPCs and the specific macro-areas of birth compared to native-born Italians. Basic models were adjusted only for age (continuous) and sex, while fully adjusted models were additionally adjusted for the whole set of covariates. For TC and LDL-C, a random intercept was added to the models, running linear regression mixed models (LMMs) to account for the laboratory in charge of the serum analyses. For the analyses of the association of migratory status with hypertension prevalence, a log link function was used in the models, and prevalence ratios (PRs) were calculated. Estimates and 95% confidence intervals (95% CI) were reported.

Each HMPC category based on age at arrival and length of stay in Italy was then compared to native-born Italians, using the same previously defined models for each outcome. This analysis was also conducted for each of the three macro-areas with sufficient sample sizes (CE Europe, Northern Africa, Asia).

All analyses were stratified by sex.

Analyses were performed using the statistical software Stata/SE version 13.0 (Stata Corp LP, College Station, TX, USA) and R (R Development Core Team 2010, R Foundation for Statistical Computing, Vienna, Austria. ISBN 3-900051-07-0, URL: http://www.R-project.org/). We employed the “lme4” and “prLogistic” packages to run LMMs and calculate prevalence ratios, respectively.

### 2.6. Ethical aspects

The study was conducted according to the guidelines of the Declaration of Helsinki and approved by the Regional (Veneto Region) Ethics Committee (24 maggio 2017 prot. No. 203638). Informed consent was obtained from all subjects involved in the study.

## 3. Results

### 3.1. Characteristics of studied populations according to immigrant status

Overall, 37,380 subjects were included in the analysis, and 3,249 (8.7%) of them were born in an HMPC. Half of the immigrants came from CE Europe, 20.1% from Northern Africa, 17.7% from Asia, 6.1% from CS America and 6% from SS Africa. The characteristics of native-born Italians and immigrants are presented in Table 1. Immigrants were younger than native-born, and their educational attainment was much lower than that of their native-born counterparts, especially for males. Immigrants reported considerably higher percentages of nonsmoking and not drinking alcohol. The percentages of obese subjects were higher among subjects born in an HMPC than among native-born Italians.

**Table 1:** Characteristics of the included subjects by immigrant status.

**Characteristics**		**HMPC (*****n*** = **3,249)**						**Italy (*****n*** = **34,131)**					
		**Total**		**Males (*****n*** = **1,317)**		**Females (*****n*** = **1,932)**		**Total**		**Males (*****n*** = **16,493)**		**Females (*****n*** = **17,638)**	
		**Median (IQR)**	**Min-Max**	**Median (IQR)**	**Min-Max**	**Median (IQR)**	**Min-Max**	**Median (IQR)**	**Min-Max**	**Median (IQR)**	**Min-Max**	**Median (IQR)**	**Min-Max**
Age (years)		40 (33–47)	20–66	41 (34–48)	20–66	39 (33–46)	20–66	42 (32–50)	20–68	42 (31–50)	20–67	43 (32–51)	20–68
		*N*	%	*N*	%	*N*	%	*N*	%	*N*	%	*N*	%
BMI	Normal weight	1,464	45.1%	510	38.7%	954	49.4%	19,620	57.5%	7,762	47,1%	11,858	67,2%
	Overweight	1,154	35.5%	574	43.6%	580	30.0%	10,103	29.6%	6,388	38,7%	3,715	21,1%
	Obese	631	19.4%	233	17.7%	398	20.6%	4,408	12.9%	2,343	14,2%	2,065	11,7%
Smoking habit	Non-smoker	2,211	68.1%	747	56.7%	1,464	75.8%	19,917	58.4%	8,101	49,1%	11,816	67,0%
	Current-smoker	593	18.3%	301	22.9%	292	15.1%	7,634	22.4%	4,444	26,9%	3,190	18,1%
	Previous smoker	445	13.7%	269	20.4%	176	9.1%	6,580	19.3%	3,948	23,9%	2,632	14,9%
Alcohol intake	None	1,700	52.3%	564	42.8%	1,136	58.8%	9,074	26.6%	2,260	13,7%	6,814	38,6%
	1–2	826	25.4%	291	22.1%	535	27.7%	11,892	34.8%	5,017	30,4%	6,875	39,0%
	3+	723	22.3%	462	35.1%	261	13.5%	13,165	38.6%	9,216	55,9%	3,949	22,4%
Education	Elementary/Middle	1,586	48.8%	725	55.0%	861	44.6%	10,531	30.9%	5,204	31,6%	5,327	30,2%
	Highschool	1,353	41.6%	505	38.3%	848	43.9%	17,313	50.7%	8,780	53,2%	8,533	48,4%
	University	310	9.5%	87	6.6%	223	11.5%	6,287	18.4%	2,509	15,2%	3,778	21,4%
Laboratory	Arzignano	1,880	57.9%	798	60.6%	1,082	56.0%	19,792	58.0%	9,622	58,3%	10,170	57,7%
	Legnago	771	23.7%	320	24.3%	451	23.3%	6,527	19.1%	3,130	19,0%	3,397	19,3%
	San bonifacio	598	18.4%	199	15.1%	399	20.7%	7,812	22.9%	3,741	22,7%	4,071	23,1%
Central-Eastern Europe	1,628	50.1%	569	43.2%	1,059	54.8%						
Sub-Saharan Africa	196	6.0%	105	8.0%	91	4.7%						
Northern Africa	652	20.1%	303	23.0%	349	18.1%						
Asia	576	17.7%	279	21.2%	297	15.4%						
Central-Southern America	197	6.1%	61	4.6%	136	7.0%						
Age at arrival (years)^*^	<18	495	16.1%	222	18.0%	273	14.9%						
	≥18	2,571	83.9%	1,015	82.1%	1,556	85.1%						
Length of stay in Italy (years)^*^	0–9	458	14.9%	131	10.6%	327	17.9%						
	10–19	1,795	58.5%	682	55.1%	1,113	60.9%						
	20+	813	26.5%	424	34.3%	389	21.3%						

^*^These variables were calculated for a subgroup of HMPC subjects, after excluding incompleted residential histories.

We retrieved the year of immigration from 94.7% of all immigrants (3,066 subjects). Over half of them resided in Italy for between 10 and 19 years (58.6%), and the majority of all immigrants were younger than 18 years of age upon their arrival in Italy (83.9%) (Supplementary Table S1).

### 3.2. Associations between immigrant status and cardiovascular risk factors

Table 2 provides estimates (β coefficients) and 95% confidence intervals (95% CI) for models assessing the associations between immigrant status and the selected cardiovascular risk factors.

**Table 2:** β coefficients and 95% confidence intervals for the associations between immigrant status and total cholesterol, LDL cholesterol, systolic blood pressure and diastolic blood pressure.

	**Total cholesterol**		**LDL cholesterol**	

	β**1 (95% CI)**	β**2 (95% CI)**	β**1 (95% CI)**	β**2 (95% CI)**
**Italy**	138.93	137.02	59.83	56.33
HMPC overall	0.2 (−1.03; 1.43)	0.23 (−1.03; 1.5)	**1.96** (0.86; 3.06)	1.09 (−0.04; 2.22)
Central-Eastern Europe	**4.39** (2.69; 6.09)	**3.31** (1.61; 5.01)	**5.90** (4.37; 7.42)	**4.50** (2.98; 6.02)
Sub-Saharan Africa	−4.7 (−9.43; 0.04)	**−5.00** (−9.73; −0.28)	−2.17 (−6.41; 2.07)	−3.57 (−7.78; 0.65)
Northern Africa	**−8.64** (−11.27; −6.01)	**−7.69** (−10.4; −4.99)	**−5.20** (−7.55; −2.84)	**−6.19** (−8.61; −3.77)
Asia	−0.43 (−3.24; 2.38)	0.85 (−2.01; 3.71)	1.25 (−1.27; 3.77)	1.02 (−1.54; 3.57)
South America	2.17 (−2.59; 6.93)	1.39 (−3.32; 6.11)	−0.21 (−4.48; 4.05)	−1.02 (−5.23; 3.18)
	**Systolic blood pressure**		**Diastolic blood pressure**	
	β**1 (95% CI)**	β**2 (95% CI)**	β**1 (95% CI)**	β**2 (95% CI)**
**Italy**	109.17	105.61	64.76	65.28
HMPC overall	−0.46 (−0.99; 0.07)	**−1.27** (−1.81; −0.72)	−0.2 (−0.56; 0.16)	**−0.52** (−0.88; −0.15)
Central-Eastern Europe	−0.48 (−1.21; 0.26)	**−1.35** (−2.08; −0.63)	0.19 (−0.3; 0.69)	−0.24 (−0.73; 0.25)
Sub-Saharan Africa	0.53 (−1.64; 2.71)	−0.27 (−2.4; 1.86)	0.02 (−1.45; 1.49)	−0.43 (−1.86; 1.01)
Northern Africa	−0.09 (−1.19; 1.02)	**−1.23** (−2.36; −0.1)	**−1.06** (−1.81; −0.31)	**−1.49** (−2.25; −0.73)
Asia	−1.02 (−2.23; 0.19)	−1.21 (−2.43; 0.01)	−0.22 (−1.04; 0.6)	−0.1 (−0.92; 0.72)
South America	−0.95 (−2.98; 1.08)	−1.64 (−3.62; 0.34)	−0.57 (−1.94; 0.8)	−1.02 (−2.35; 0.31)

β1, basic adjustment, adjusting for only age and sex; β2, full adjustment, adjusting for the whole set of covariates. Statistically significant associations are displayed in bold.

In basic-adjusted models (adjusted for age and sex), no significant differences were observed for the TC levels between foreign-born adults and their native-born counterparts, while the LDL-C levels of immigrants were significantly higher than those of native-born Italians. Considering the results for each geographical macro-area of origin, immigrants from CE Europe had significantly higher TC and LDL-C levels than native-born Italians, while immigrants from Northern Africa showed significantly lower levels of both TC and LDL-C than native-born Italians. The higher levels of LDL-C among immigrants disappeared in the fully adjusted model (1.09 mg/dl, 95% CI: −0.04; 2.22). Effect estimates on cholesterol levels for each geographical macro-area with the full adjustment were similar to those unadjusted, although slightly attenuated. Immigrants from CE Europe had significantly higher levels of both TC and LDL-C (TC: 3.31 mg/dl, 95% CI: 1.61; −5.01, LDL-C: 4.50 mg/dl, 95% CI: 2.98; −6.02), and immigrants from Northern Africa had significantly lower levels of both TC and LDL-C (TC: −7.69 mg/dl, 95% CI: −10.4; −4.99, LDL-C: −6.19 mg/dl, 95% CI: −8.61; −3.77).

No significant difference was observed for BP levels between foreign-born adults and their native-born counterparts in basic adjusted models. In fully adjusted models, immigrants overall had significantly lower levels of SBP and DBP (SBP: −1.27 mmHg, 95% CI: −1.81; −0.72, DBP: −0.52 mmHg, 95% CI: −0.88; −0.15) than native-born Italians. Significantly lower levels of BP were shown for the subgroups of immigrants from Northern Africa (SBP and DBP) and CE Europe (SBP only).

**Figure 1:**
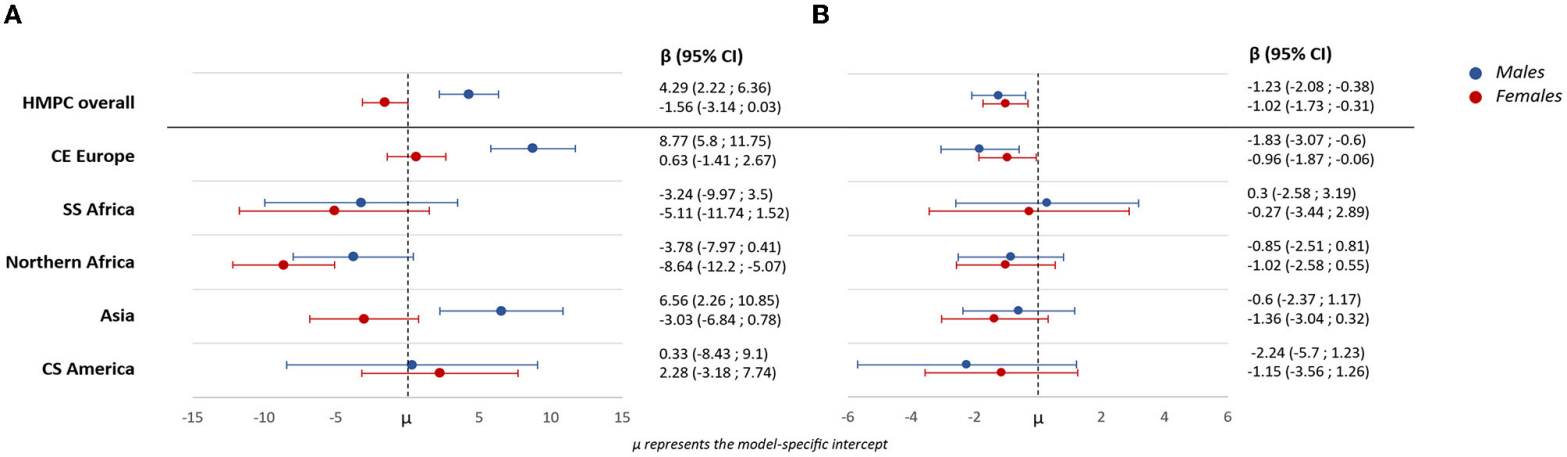
β estimates and 95% confidence intervals of the associations between country of birth (overall and by macro-areas) and total cholesterol **(A)** and systolic blood pressure **(B)**, stratified by sex and adjusted for the full set of covariates.

Figure 1 presents β estimates and 95% CI results for the associations between immigrant status (overall and by macro-areas) and TC (Panel a) and SBP (Panel b), stratified by sex and adjusted for the full set of covariates. Immigrant males had significantly higher levels of TC than native-born Italian males (4.29 mg/dl, 95% CI: 2.22; −6.36). Significantly higher levels also were seen for males from CE Europe (8.77 mg/dl, 95% CI: 5.8; −11.75) and from Asia (6.56 mg/dl, 95% CI: 2.26; −10.85). In contrast, females from Northern Africa had significantly lower levels of TC than native-born Italian females (-8.64 mg/dl, 95% CI: −12.2; −5.07).

Male and female immigrants, overall and from CE Europe, had lower levels of SBP than their native-born Italian counterparts. No significant difference was seen for immigrants from other macro-areas when stratified by sex.

Supplementary Table S2 presents the results of the prevalence ratio for hypertension in the basic and fully adjusted models, overall and stratified by sex. The results confirmed the findings observed for blood pressure levels.

### 3.3. Associations between immigrant status and cardiovascular risk factors in relation to the migratory pathway

**Figure 2:**
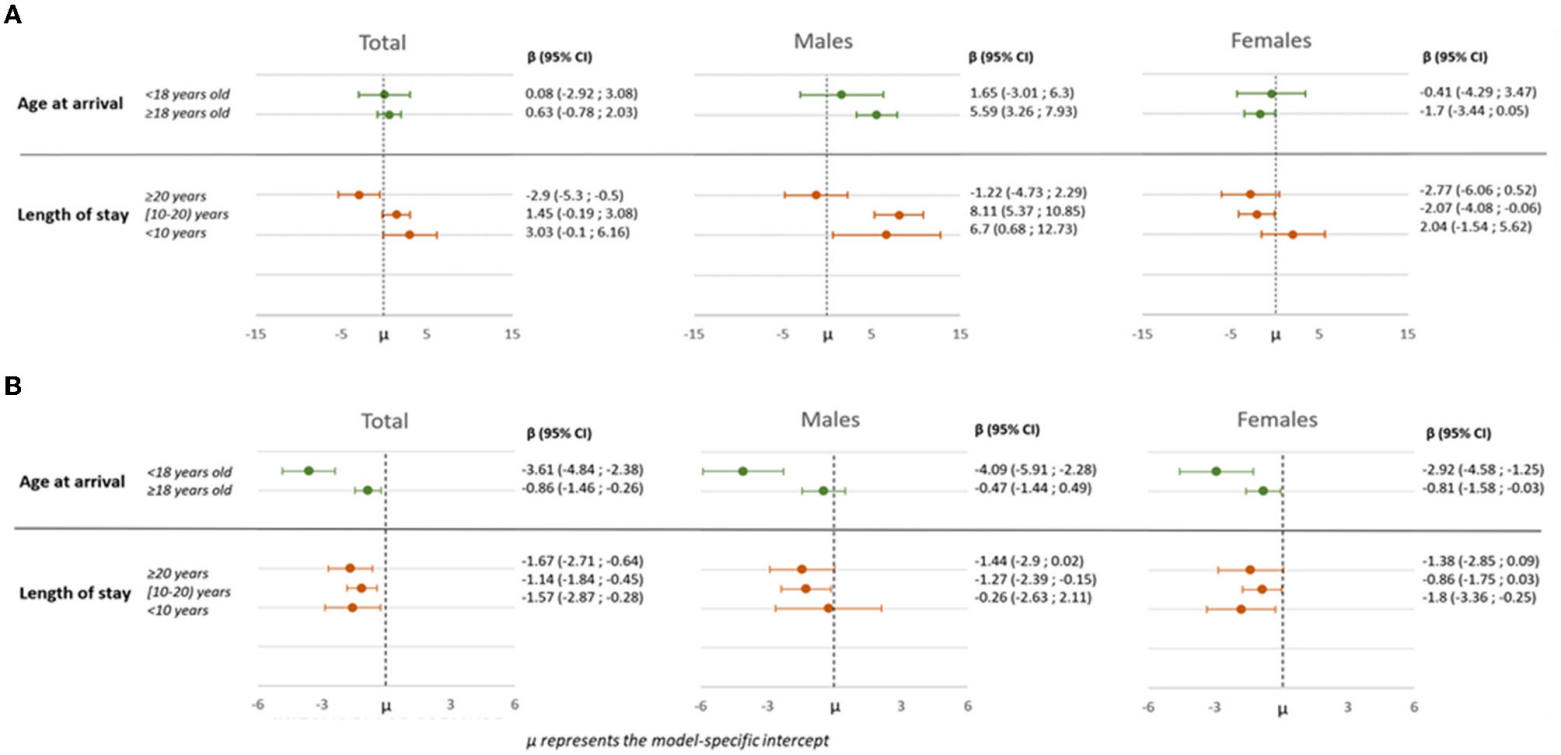
β estimates and 95% confidence intervals of the associations between country of birth and total cholesterol **(A)** and systolic blood pressure **(B)** in relation to duration of residence and age at migration by sex.

Figures 2A, B shows the association between country of birth and TC and SBP in relation to duration of residence and age at migration by sex.

Immigrants who resided for <10 years in Italy had higher levels of TC compared to their native-born Italian counterparts. This difference gradually decreased with a longer stay, reaching significantly lower levels of TC for those who lived in Italy for more than 20 years. This trend was particularly clear for males, with those living in Italy <20 years showing higher levels of TC than Italian-born adults. Among females, the trend was less clear, with significantly lower levels only for those who resided in Italy between 10 and 20 years. Additionally, considering age at arrival, differences were seen for males only, with higher TC levels for males who arrived in Italy and were older than 18 years compared to native-born Italians (Panel a).

When the macro-areas of origin were considered (Supplementary Table S3), immigrants from CE Europe and Asia had patterns similar to the overall pattern. For immigrants from CE Europe, higher levels of TC also were seen for those who arrived older than 18 years. In contrast, immigrants from Northern Africa showed an opposite trend: their advantage eroded with an increasing stay in the country and with a younger age at arrival.

Immigrants who arrived under 18 years old had lower levels of SBP compared to native-born Italians, overall and for both sexes. This advantage persisted but was reduced for subjects who arrived after 18 years of age. The same advantage was seen in all groups when duration of residence was considered, but with a less clear gradient (Figure 2B). The greater advantage of those who arrived younger is maintained in subjects coming from all macro-areas of origin (Supplementary Table S3).

## 4. Discussion

In our study, immigrant adult males showed higher cholesterol levels than their native-born Italian counterparts, while both immigrant males and females had lower BP levels. The higher levels of TC are mainly driven by the subjects from CE Europe, representing more than 50% of all immigrants. Immigrant males from Asia had higher levels of TC, while immigrants from Northern Africa showed lower levels of cholesterol than native-born Italians, although the results were significant for immigrant females only. Immigrants from CE Europe and Northern Africa had lower levels of SBP than native-born Italians.

After adjustments for known determinants (lifestyle factors and social determinants) of CV risk factors, the results maintained the same direction, although differences were attenuated in some cases and increased in other cases. These differences probably depend on the deep heterogeneity of the lifestyle behaviors of immigrants from different macro-areas. Regardless, the preserved direction in the adjusted results suggests that these results are only partially driven by environmental factors of the host country. Genetic factors and early living conditions can play a role in explaining these differences. The prevalence of CV risk factors, especially a high-calorie diet and cardiovascular disease, is high in Eastern Europe (16). Therefore, when immigrants from these countries arrive in Italy, they already have a background of worse health behaviors than the native population. This could explain the higher levels of TC in subjects from CE Europe. In contrast, the reduced BP levels in immigrants from CE Europe are less expected. Our results are in contrast with the European estimates of BP levels, which indicate a 10% higher prevalence of raised BP in Albania and Romania (the two most represented countries of birth in our sample; see Supplementary Table S4) than in Italy in 2014 (16). A study conducted in the same Italian region found results similar to our study, with Italian citizens having the highest rates of hypertension compared to all groups of immigrants except those coming from SS Africa (5). Regardless, it should be noted that the differences in BP levels between immigrants and natives are small in magnitude and that no differences were seen between immigrants from CE Europe and native-born Italians when considering the adjusted results for the prevalence of hypertension.

A distinct pattern of cardiovascular risk could be identified among Asians, with higher rates of TC among males than among their native-born Italian counterparts. When interpreting the results of Asian immigrants, the composition within this category is worth noting. In fact, 85% of this population in our study consisted of subjects born in the Indian subcontinent (Supplementary Table S4). The findings of our study are consistent with previous reports on ethnic differences in plasma lipid levels in the United States, Canada, the United Kingdom and the Netherlands, which have shown that South Asians are characterized by high LDL-C and triglyceride levels and low HDL-C compared to the reference population (10, 17–19). The reasons for this unfavorable lipid pattern seem to be both environmental and genetic. It has been demonstrated that South Asians in the US were less physically active and had lower adiponectin and higher resistin levels than Caucasians, resulting in higher levels of LDL-C (20).

Regarding immigrants from Northern Africa, in our cohort, they are mostly represented by subjects born in Morocco (96%, see Supplementary Table S4). The low prevalence of dyslipidemia in Moroccans is consistent with the low LDL-C and TG levels found by Gazzola et al. in Moroccan descendants compared to the Dutch reference population (10). Additionally, Moroccans are traditionally known to have a lower prevalence of hypertension in Europe, although this health advantage seems to be changing unfavorably through the acculturation process (21). The favorable lifestyle and behavioral habits that are linked with the Muslim population (highly prevalent in Morocco) may represent an advantage reflecting a lower predisposition toward the development of dyslipidemia and hypertension. Differences in BP levels related to the prevalent religion have been previously found (11), and such an explanation also could be valid for the lipid pattern.

Our results on cholesterol also showed relevant gender differences: males from CE Europe and Asia had higher TC levels than native-born Italians, while no differences were seen for their female counterparts. In contrast, females from Northern Africa had lower levels of TC than native-born Italians, and this difference was less clear for males. Additionally, the trends related to acculturation, with results approaching those of the native population in immigrants with a longer stay, are stronger in males than in females. These differences may depend on the prevalence of risky behaviors: smoking and alcohol intake are more prevalent in immigrant males than in females in our population. Additionally, in the group of immigrants from CE Europe, the rate of overweight or obese people is doubled among males compared with females. This is not true for immigrants from Northern Africa, though. Additionally, these differences could be related to different approaches toward health care. In a Dutch study, women were found to have higher levels of awareness, treatment and control of hypertension than men in all ethnic groups, and this has been attributed to frequent use of health care by women (21, 22).

Although environmental and socioeconomic factors play only partial roles in determining these disparities, an effort should be made to reduce the risky health behaviors and potential mediators of these differences. Many interventions in primary health care settings have been demonstrated to be effective for the early prevention of cardiovascular diseases. In particular, the higher BMI of the immigrant population could be reduced with interventions on nutritional habits and physical activity. Additionally, reduced access to primary health care (PHC) could lead to a later diagnosis and a later start of adequate therapy. Immigrants from HMPCs have a probability of an annual LDL-C test reduced by half compared to native-born Italians (12). PHC services in Italy are, in theory, widely accessible and mostly free at the point of use; however, different economic resources might provide access to more timely private services, and different levels of education might affect people's approaches when managing their health problems (23). Furthermore, the cultural backgrounds and health practices of immigrants may be dissimilar to those of European people and health professionals, and it is essential to take particular care when dealing with these patients.

A clear gradient was seen for what concerns the levels of cholesterol by length of stay: immigrants who stayed longer than 20 years in Italy also had lower levels of TC than native-born Italians, while those who arrived <10 years ago had similar or higher levels of TC compared to native-born Italians. Additionally, immigrants who arrived younger than 18 years had lower levels of SBP than native-born Italians, and these results were attenuated in those who arrived older than 18 years. These results, too, were driven by the large group of immigrants from CE Europe in our population. Several studies suggest that acculturation is associated with a decline in healthy behaviors, resulting in an increase in CV risk factors (24, 25), although the evidence concerning the relationships among acculturation, lifestyle behaviors, and cardiovascular risk factors is not uniform, with some examples of convergence from initially higher levels of risk factors down to local levels (26–28). These different findings mainly depend on the ethnic groups considered (29) and the starting conditions in their country of origin. In our study, the acculturation of immigrants from CE Europe seems to be a protective factor against CV risk factors. As previously said, this could reflect the higher prevalence of the aforementioned CV risk factors in CE Europe. A longer stay in Italy, as well as an arrival at a younger age, could lead to an earlier and longer adoption of healthy behavior and result in lower CV risk factors. For other immigrant groups, the process of acculturation could lead to an increased risk. The health advantage that was seen for immigrants from Northern Africa, with lower levels of TC compared to native-born Italians, declines with an increasing length of stay and disappears for immigrants who arrived younger than 18 years. In this case, the obesogenic environment of the host country probably plays a major role in changing the virtuous lifestyle habits of immigrants. For the Asian group, the results are more heterogeneous, and it is difficult to draw any conclusion concerning this specific category.

To our knowledge, this is one of the first studies conducted in Italy analyzing differences in biochemical and clinical parameters among native-born Italians and immigrants and the first that evaluates the effect of the migratory pathway in modifying these outcomes. It has been conducted on a large number of individuals and accounts for a wide number of potential characteristics associated with CV risk factors. The results also pave the way to future analysis considering other CV risk factors, such as glycated hemoglobin as indicator of the risk of developing diabetes mellitus. This could be an interesting point, to early identify subjects and groups at risk, and to tailor interventions before the development of the disease itself, which has been shown to be more prevalent in immigrants than natives (12). The study also has some limitations. First, we do not know the response rates of immigrants and natives. The questionnaire was conducted in Italian, and it was very complex, so there could have been a selection bias in the responders. The questionnaires were administered by trained nurses and not self-administered, so nurses could have mediated some language barriers by explaining the questions. Additionally, when communication was hindered by language problems, the interviewed subject was invited to return a second time with an interpreter. Second, we do not have information on the citizenship of the responders, and the “immigrant” category was built on the country of birth alone. Therefore, we could have included in this category Italian citizens but incidentally born abroad, although it is probably a quite exceptional occurrence when considering developing countries. Third, the Surveillance Program does not include people born before 1951, and this study therefore does not include elderlies. This limitation can be relevant in the analysis with hypertension as outcome, since the prevalence of this disease raises with age, although it must be pointed out that the prevalence of immigrant subjects in Italy of this age group is minimal.

In conclusion, it is clear that immigrants should not be considered a homogenous group when exploring their health outcomes. Tailored prevention and follow-up programs are required to address differences and to target the worse-off groups. Immigrants from CE Europe, especially those who arrived at older ages, arrive with a health disadvantage compared to native-born Italians, and programs raising awareness of worse habits can be useful to accelerate the process of acculturation. In contrast, immigrants from Northern Africa have a health advantage that should be preserved to avoid future increases in CV diseases.

## Data availability statement

The raw data supporting the conclusions of this article will be made available by the authors, without undue reservation.

## Ethics statement

The study was approved by Regional (Veneto Region) Ethics Committee (24 maggio 2017 prot. No. 203638). Written informed consent for participation was obtained from all subjects involved in the study.

## Author contributions

TDZ wrote the manuscript, discussed, and interpreted the results of the data. EB performed the statistical analysis. TS, FR, and GP reviewed and edited the manuscript. CC is the guarantor of this work and, as such, had full access to all the data in the study and takes responsibility for the integrity of the data, and the accuracy of the data analysis. All authors have read and agreed to the published version of the manuscript.

## Supplementary material

Supplementary Figure S1 Flowchart of participants included in the study.
