# Supplementary figures and images for: Adult first-generation immigrants and cardiovascular risk factors in the Veneto Region, Northeast Italy
Source: Front Public Health. 2023 Feb 15;11:956146. doi: 10.3389/fpubh.2023.956146 (PMC9975734; doi:10.3389/fpubh.2023.956146)

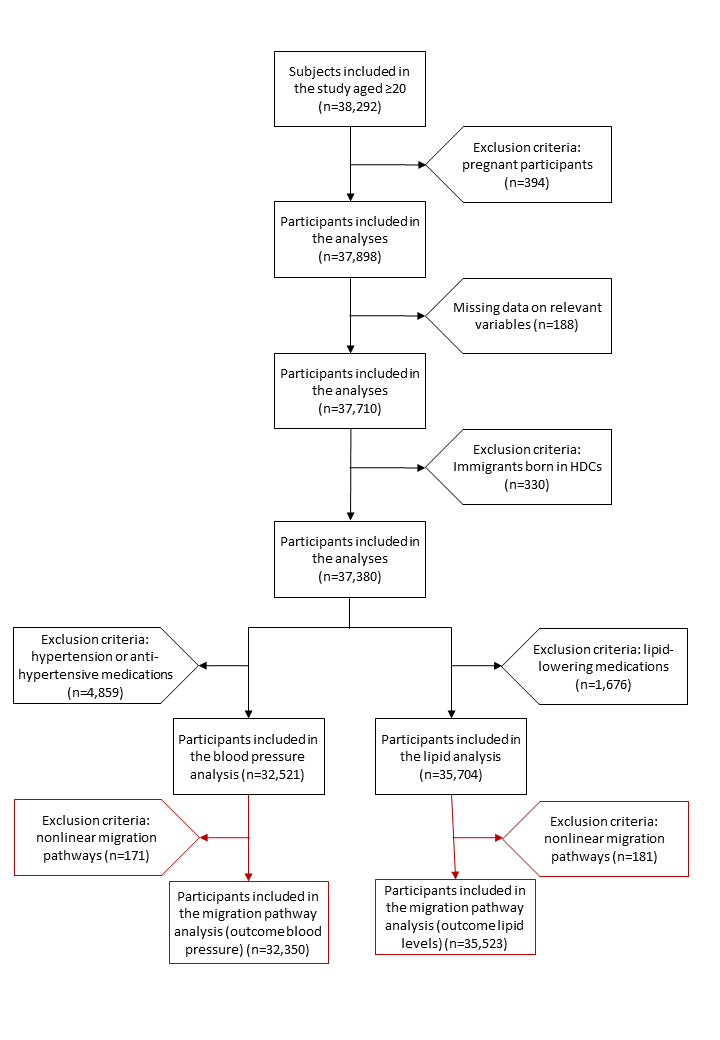

Supplement: Supplementary Figure S1 — Flowchart of participants included in the study. [file Image_1.png]
