# Supplementary material for: Adult first-generation immigrants and cardiovascular risk factors in the Veneto Region, Northeast Italy
Source: Front Public Health. 2023 Feb 15;11:956146. doi: 10.3389/fpubh.2023.956146 (PMC9975734; doi:10.3389/fpubh.2023.956146)
Supplement: Supplementary file 2 [file Data_Sheet_1.pdf]

Supplementary Material

1 Supplementary Figures

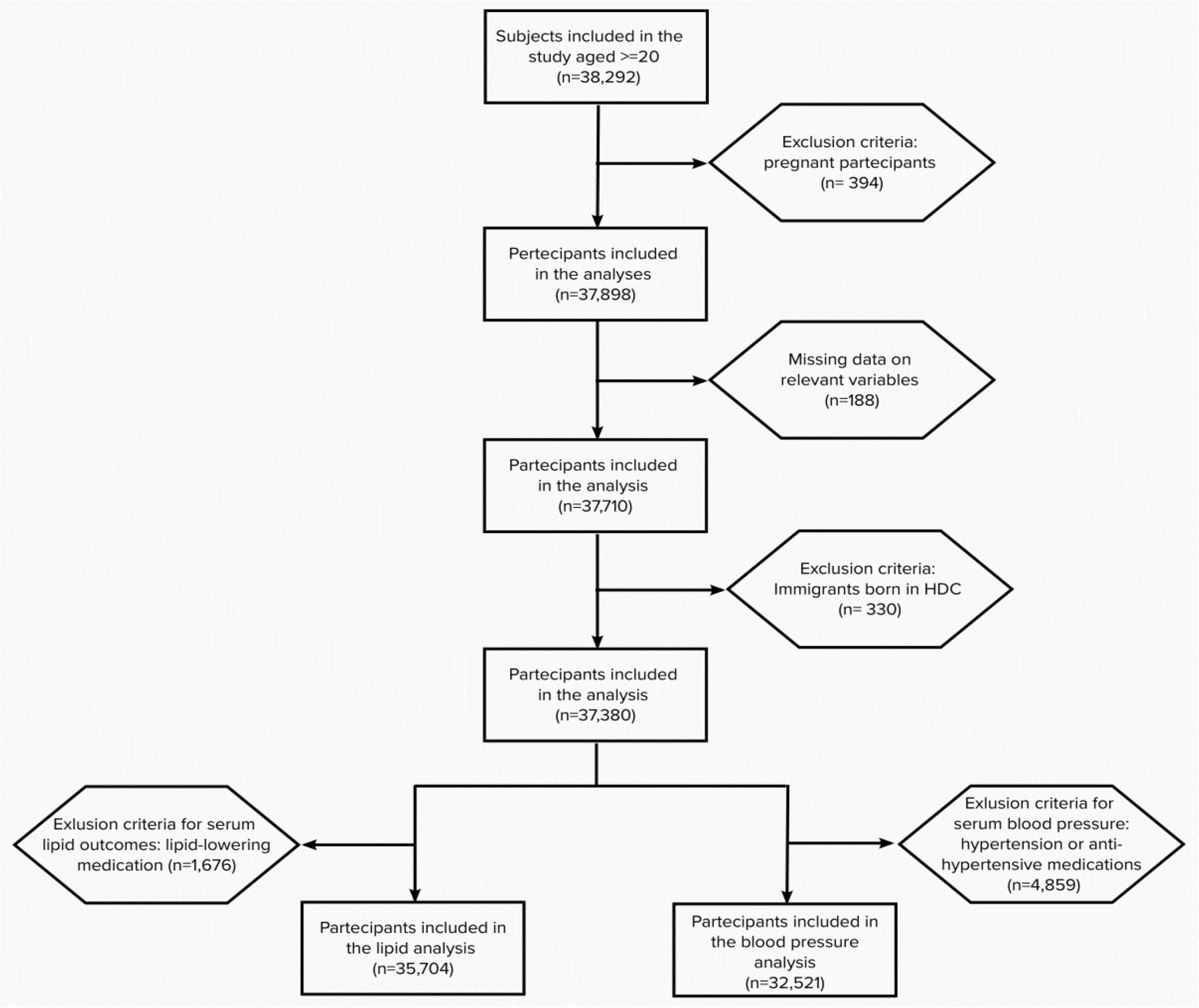

Supplementary Figure 1. Flowchart of participants included in the study. (see the attached .png file for the full resolution)

2 Supplementary Tables

Characteristics (n=3066)		ASIA (n=558)						North Africa (n=630)						CE Europe (n=1525)						South Africa (n=185)						South America (n=168)					
		Total		Males		Females		Total		Males		Females		Total		Males		Females		Total		Males		Females		Total		Males		Females	
		Median (IQR)	*Min-Max*	*Median (IQR)*	*Min-Max*	*Median (IQR)*	*Min-Max*	*Median (IQR)*	*Min-Max*	*Median (IQR)*	*Min-Max*	*Median (IQR)*	*Min-Max*	*Median (IQR)*	*Min-Max*	*Median (IQR)*	*Min-Max*	*Median (IQR)*	*Min-Max*	*Median (IQR)*	*Min-Max*	*Median (IQR)*	*Min-Max*	*Median (IQR)*	*Min-Max*	*Median (IQR)*	*Min-Max*	*Median (IQR)*	*Min-Max*	*Median (IQR)*	*Min-Max*
Age (years)		39 (33-46)	20-65	41 (33-48)	20-64	38 (32-45)	20-65	41 (34-47)	20-66	43 (37-49)	20-65	39 (32-45)	20-66	40 (34-47)	20-66	41 (34-48)	20-66	40 (34-47)	20-66	40 (33-47)	20-61	43 (34-50)	21-61	38 (32-45)	20-56	41.5 (33-49)	20-65	40 (32-47)	21-62	42 (33-49)	20-65
Age at arrival (years)		26 (21-31)	0-55	25 (20-30)	0-55	26 (22-32)	0-55	24 (19-29)	0-49	25 (21-29)	0-43	23 (19-29)	0-49	24 (20-30)	0-55	25 (20-30)	0-53	24 (20-31)	0-55	26 (20-30)	0-40	25.5 (20-28)	0-47	15 (10-21)	2-49	23.5 (12-29)	0-48	20 (9-26)	0-47	24 (17-30)	0-48
Length of Stay in Italy (years)		13 (10-18)	1-56	16 (11-20)	1-56	11 (8-15)	1-50	17 (12-21)	1-60	18 (13-22)	1-50	15 (11-20)	1-60	16 (12-19)	0-59	17 (13-21)	0-41	15 (12-18)	1-59	26 (20-29)	0-47	16 (10-25)	2-46	14 (9-19)	2-49	18 (13-26)	3-60	19 (15-28)	8-60	17 (12-24)	3-46
% of life spent in Italy (years %)		34.9 (25.0-43.9)	2.6-100	40 (31.3-47.5)	2.6-100	30 (21.8-38.1)	4.6-100	40.9 (31.3-51.9)	3.1-100	43.2 (32.8-53.7)	3.1-100	39.2 (30-50)	4.6-100	38.7 (30.6-47.1)	0-100	40.9 (32.8-50)	0-100	37.7 (29.4-46.2)	3.7-100	37.5 (27.6-50)	5.7-100	39.0 (28.1-50.9)	5.7-100	37.0 (26.3-44.7)	7.8-100	44.7 (33.3-64.4)	7.7-100	51.2 (37.5-71.8)	23.0-100	41.2 (31.8-58.8)	7.7-100
		N	*%*	*N*	*%*	*N*	*%*	*N*	*%*	*N*	*%*	*N*	*%*	*N*	*%*	*N*	*%*	*N*	*%*	*N*	*%*	*N*	*%*	*N*	*%*	*N*	*%*	*N*	*%*	*N*	*%*
BMI	Normal weight	251	45.0%	121	44.5%	130	45.5%	248	39.4%	142	48.5%	106	31.5%	712	46.7%	150	28.5%	562	56.3%	76	41.1%	47	47.5%	29	33.7%	94	56.0%	17	36.2%	77	63.6%
	Overweight	215	38.5%	115	42.3%	100	35.0%	234	37.1%	111	37.9%	123	36.5%	527	34.6%	256	48.7%	271	27.1%	63	34.1%	35	35.4%	28	32.6%	51	30.4%	23	48.9%	28	23.1%
	Obese	92	16.5%	36	13.2%	56	19.6%	148	23.5%	40	13.7%	108	32.0%	286	18.8%	120	22.8%	166	16.6%	46	24.9%	17	17.2%	29	33.7%	23	13.7%	7	14.9%	16	13.2%
Smoking Habit	Non-smoker	470	84.2%	196	72.1%	274	95.8%	508	80.6%	181	61.8%	327	97.0%	845	55.4%	223	42.4%	622	62.3%	159	85.9%	76	76.8%	83	96.5%	115	68.5%	27	57.4%	88	72.7%
	Current-smoker	57	10.2%	48	17.6%	9	3.1%	67	10.6%	59	20.1%	8	2.4%	383	25.1%	150	28.5%	233	23.3%	14	7.6%	13	13.1%	1	1.2%	28	16.7%	9	19.1%	19	15.7%
	Previous smoker	31	5.6%	28	10.3%	3	1.0%	55	8.7%	53	18.1%	2	0.6%	297	19.5%	153	29.1%	144	14.4%	12	6.5%	10	10.1%	2	2.3%	25	14.9%	11	23.4%	14	11.6%
Alcohol intake	None	397	71.1%	142	52.2%	255	89.2%	593	94.1%	266	90.8%	327	97.0%	482	31.6%	75	14.3%	407	40.7%	107	57.8%	53	53.5%	54	62.8%	49	29.2%	5	10.6%	44	36.4%
	1-2	85	15.2%	60	22.1%	25	8.7%	19	3.0%	14	4.8%	5	1.5%	544	35.7%	147	27.9%	397	39.7%	48	25.9%	24	24.2%	24	27.9%	58	34.5%	19	40.4%	39	32.2%
	3+	76	13.6%	70	25.7%	6	2.1%	18	2.9%	13	4.4%	5	1.5%	499	32.7%	304	57.8%	195	19.5%	30	16.2%	22	22.2%	8	9.3%	61	36.3%	23	48.9%	38	31.4%
Education	Elementary/Middle	338	60.6%	177	65.1%	161	56.3%	466	74.0%	211	72.0%	255	75.7%	550	36.1%	227	43.2%	323	32.3%	115	62.2%	58	58.6%	57	66.3%	58	34.5%	21	44.7%	37	30.6%
	Highschool	162	29.0%	75	27.6%	87	30.4%	125	19.8%	59	20.1%	66	19.6%	821	53.8%	273	51.9%	548	54.9%	56	30.3%	36	36.4%	20	23.3%	83	49.4%	20	42.6%	63	52.1%
	University	58	10.4%	20	7.4%	38	13.3%	39	6.2%	23	7.8%	16	4.7%	154	10.1%	26	4.9%	128	12.8%	14	7.6%	5	5.1%	9	10.5%	27	16.1%	6	12.8%	21	17.4%
Laboratory	Arzignano	398	71.3%	203	74.6%	195	68.2%	258	41.0%	128	43.7%	130	38.6%	982	64.4%	361	68.6%	621	62.2%	107	57.8%	62	62.6%	45	52.3%	89	53.0%	23	48.9%	66	54.5%
	Legnago	121	21.7%	53	19.5%	68	23.8%	197	31.3%	90	30.7%	107	31.8%	303	19.9%	107	20.3%	196	19.6%	41	22.2%	24	24.2%	17	19.8%	50	29.8%	17	36.2%	33	27.3%
	San Bonifacio	39	7.0%	16	5.9%	23	8.0%	175	27.8%	75	25.6%	100	29.7%	240	15.7%	58	11.0%	182	18.2%	37	20.0%	13	13.1%	24	27.9%	29	17.3%	7	14.9%	22	18.2%
Age at arrival (years)	<18	78	14.0%	45	16.5%	33	11.5%	124	19.7%	53	18.1%	71	21.1%	217	14.2%	90	17.1%	127	12.7%	26	14.1%	15	15.2%	11	12.8%	50	29.8%	19	40.4%	31	25.6%
	≥18	480	86.0%	227	83.5%	253	88.5%	506	80.3%	240	81.9%	266	78.9%	1308	85.8%	436	82.9%	872	87.3%	159	85.9%	84	84.8%	75	87.2%	118	70.2%	28	59.6%	90	74.4%
Length of Stay in Italy (years)	0-9	133	23.8%	45	16.5%	88	30.8%	81	12.9%	25	8.5%	56	16.6%	181	11.9%	38	7.2%	143	14.3%	45	24.3%	22	22.2%	23	26.7%	18	10.7%	1	2.1%	17	14.0%
	10-19	315	56.5%	148	54.4%	167	58.4%	342	54.3%	156	53.2%	186	55.2%	975	63.9%	315	59.9%	660	66.1%	85	45.9%	40	40.4%	45	52.3%	78	46.4%	23	48.9%	55	45.5%
	20+	110	19.7%	79	29.0%	31	10.8%	207	32.9%	112	38.2%	95	28.2%	369	24.2%	173	32.9%	196	19.6%	55	29.7%	37	37.4%	18	20.9%	72	42.9%	23	48.9%	49	40.5%
% of life spent in Italy (years %)	0%-24%	132	23.7%	38	14.0%	94	32.9%	83	13.2%	31	10.6%	52	15.4%	189	12.4%	41	7.8%	148	14.8%	36	19.5%	17	17.2%	19	22.1%	23	13.7%	3	6.4%	20	16.5%
	25%-49%	351	62.9%	184	67.6%	167	58.4%	363	57.6%	169	57.7%	194	57.6%	1029	67.5%	351	66.7%	678	67.9%	102	55.1%	54	54.5%	48	55.8%	73	43.5%	17	36.2%	56	46.3%
	≥50%	75	13.4%	50	18.4%	25	8.7%	184	29.2%	93	31.7%	91	27.0%	307	20.1%	134	25.5%	173	17.3%	47	25.4%	28	28.3%	19	22.1%	72	42.9%	27	57.4%	45	37.2%

Supplementary table 1. Characteristics of the included subjects by macro-area of origin

	HYPERTENSION					
	TOTAL		MALES		FEMALES	
	**Basic-adj PR (95% CI)**	**Full-adj PR (95% CI)**	**Basic-adj PR (95% CI)**	**Full-adj PR (95% CI)**	**Basic-adj PR (95% CI)**	**Full-adj PR (95% CI)**
**Italy**	1	1	1	1	1	1
HMPC overall	0.96 (0.88-1.06)	0.80 (0.72-0.88)	0.82 (0.73-0.93)	0.79 (0.69-0.90)	1.21 (1.06-1.38)	0.87 (0.75-1.00)
Central-Eastern Europe	1.10 (0.97-1.24)	0.88 (0.77-1.00)	1.05 (0.88-1.25)	0.84 (0.69-1.02)	1.19 (1.01-1.41)	0.95 (0.79-1.15)
Sub-Saharan Africa	1.42 (1.03-1.97)	1.12 (0.79-1.57)	0.92 (0.61-1.40)	0.94 (0.60-1.47)	2.93 (1.83-4.69)	1.61 (0.97-2.68)
Northern Africa	0.61 (0.49-0.75)	0.49 (0.39-0.61)	0.46 (0.34-0.61)	0.53 (0.39-0.72)	0.99 (0.72-1.35)	0.50 (0.36-0.70)
Asia	0.98 (0.79-1.21)	0.87 (0.70-1.09)	0.86 (0.66-1.12)	0.94 (0.71-1.24)	1.28 (0.93-1.77)	0.89 (0.63-1.26)
South America	0.86 (0.59-1.26)	0.78 (0.53-1.15)	0.74 (0.41-1.33)	0.66 (0.36-1.22)	0.99 (0.61-1.59)	0.87 (0.53-1.43)

Supplementary table 2. Prevalence ratio (PR) for hypertension, basic- and full-adjusted models, overall and stratified by sex

**Variables of migratory pattern**	**TOTAL CHOLESTEROL**								
	**CENTRAL EAST EUROPE**			**NORTHERN AFRICA**			**ASIA**		
Age at arrival	β	95% CI		β	95% CI		β	95% CI	
Italy	141.01			141.10			141.34		
HMPC with <18 yo at arrival	2.38	-2.07	6.83	-4.53	-10.40	1.35	2.14	-5.39	9.67
HMPC with >18 yo at arrival	**4.27**	2.38	6.16	**-7.88**	-10.94	-4.83	0.74	-2.37	3.86
Lenght of stay									
Italy	140.80			141.20			141.25		
HMPC from >20 years in Italy	-0.55	-4.08	2.98	**-5.64**	-10.31	-0.97	-3.77	-10.15	2.62
HMPC from 10-19 years in Italy	**5.27**	3.12	7.43	**-7.80**	-11.44	-4.16	1.58	-2.22	5.39
HMPC from <10 years in Italy	**5.96**	1.07	10.86	**-8.64**	-15.94	-1.34	3.27	-2.48	9.02
**Variables of migratory pattern**	**SYSTOLIC BLOOD PRESSURE**								
	**CENTRAL EAST EUROPE**			**NORTHERN AFRICA**			**ASIA**		
Age at arrival	β	95% CI		β	95% CI		β	95% CI	
Italy	108.40			108.46			108.38		
HMPC with <18 yo at arrival	**-4.74**	-6.55	-2.93	**-2.72**	-5.16	-0.28	-2.73	-5.79	0.32
HMPC with >18 yo at arrival	-0.75	-1.56	0.07	-1.03	-2.30	0.24	-0.75	-2.09	0.59
Lenght of stay									
Italy	108.24			108.43			108.41		
HMPC from >20 years in Italy	**-2.12**	-3.64	-0.60	-1.64	-3.61	0.34	-0.46	-3.30	2.37
HMPC from 10-19 years in Italy	**-1.17**	-2.09	-0.25	-1.40	-2.91	0.11	-0.09	-1.71	1.53
HMPC from <10 years in Italy	-1.17	-3.21	0.88	-0.64	-3.59	2.30	**-3.63**	-6.01	-1.25

Supplementary table 3. Association between country of birth and TC and SBP in relation to duration of residence and age at migration, by macro-area of origin.

CENTRAL-EASTERN EUROPE	N	%
ROMANIA	568	34,9%
ALBANIA	398	24,4%
SERBIA	277	17,0%
MOLDAVIA	125	7,7%
POLAND	64	3,9%
UKRAINE	43	2,6%
BULGARIA	37	2,3%
BOSNIA	36	2,2%
RUSSIA	14	0,9%
BELARUS	10	0,6%
SLOVAK REPUBLIC	10	0,6%
CROATIA	9	0,6%
HUNGARY	7	0,4%
CZECH REPUBLIC	6	0,4%
MACEDONIA	6	0,4%
YUGOSLAVIA	5	0,3%
LATVIA	3	0,2%
LITHUANIA	3	0,2%
KOSOVO	2	0,1%
SLOVENIA	2	0,1%
ESTONIA	1	0,1%
MONTENEGRO	1	0,1%
OTHER	1	0,1%
**SUB-SAHARAN AFRICA**	**N**	**%**
IVORY COAST	50	25,5%
SENEGAL	44	22,4%
NIGERIA	32	16,3%
GHANA	28	14,3%
ETHIOPIA	9	4,6%
BURKINA FASO	7	3,6%
GUINEA	4	2,0%
KENYA	3	1,5%
TOGO	3	1,5%
BURUNDI	2	1,0%
CHAD	2	1,0%
GUINEA BISSAU	2	1,0%
SOUTH AFRICA	2	1,0%
RWANDA	2	1,0%
CAMEROON	1	0,5%

CONGO	1	0,5%
GABON	1	0,5%
MADAGASCAR	1	0,5%
MALI	1	0,5%
ZAMBIA	1	0,5%
**NORTHERN AFRICA**	**N**	**%**
MOROCCO	630	96,6%
TUNISIA	10	1,5%
EGYPT	5	0,8%
ALGERIA	4	0,6%
LIBYA	3	0,5%
**ASIA**	**N**	**%**
INDIA	393	68,2%
BANGLADESH	100	17,4%
CHINA	43	7,5%
GEORGIA	14	2,4%
SRI LANKA	7	1,2%
LEBANON	3	0,5%
PHILIPPINES	2	0,3%
IRAN	2	0,3%
KAZAKHSTAN	2	0,3%
NEPAL	2	0,3%
SYRIA	2	0,3%
THAILAND	2	0,3%
ARMENIA	1	0,2%
KYRGYZSTAN	1	0,2%
PAKISTAN	1	0,2%
UZBEKISTAN	1	0,2%
**CENTRAL-SOUTHERN AMERICA**	**N**	**%**
BRAZIL	65	33,0%
ARGENTINA	40	20,3%
PERU	20	10,2%
COLOMBIA	12	6,1%
CUBA	12	6,1%
DOMINICAN REPUBLIC	12	6,1%
VENEZUELA	11	5,6%
GUATEMALA	6	3,0%
CHILE	5	2,5%
PARAGUAY	5	2,5%
MEXICO	3	1,5%

ECUADOR	2	1,0%
BOLIVIA	1	0,5%
COSTA RICA	1	0,5%
HONDURAS	1	0,5%
NICARAGUA	1	0,5%

Supplementary Table S4. Number and percentage of subjects from each country of birth in each macro-area of origin
